# Evaluation of indolicidin for inactivation of bovine alphaherpesvirus 1 in semen

**DOI:** 10.1007/s11259-026-11544-2

**Published:** 2026-09-26

**Authors:** João Paulo Yoshio Prado Cerqueira Kubota, Gabriel Henrique Santos, Taise Maria dos Anjos Oliveira, Maria Lúcia Gambarini, Keyla de Oliveira Ribeiro, Klayto José Gonçalves dos Santos, Guilherme Sastre de Souza, Maria Carolina Oliveira de Arruda Brasil, Eduardo Maffud Cilli, Esteban Nicolás Lorenzón, Guilherme Rocha Lino de Souza

**Affiliations:** 1 Escola de Veterinária e Zootecnia, Universidade Federal de Goiás, Goiânia, GO Brazil; 2 Escola de Agronomia, Universidade Federal de Goiás, Goiânia, GO Brazil; 3 Centro de Biotecnologia em Reprodução Animal (BIOTEC), Universidade Estadual de Goiás, São Luís de Montes Belos, GO Brazil; 4 Instituto de Ciências Biológicas, Universidade Federal de Goiás, Goiânia, GO Brazil; 5 Instituto de Ciências da Saúde, Universidade Federal de Jataí, Jataí, GO Brazil; 6 Instituto de Química de Araraquara, Universidade Estadual Paulista, Araraquara, SP Brasil

**Keywords:** Semen disinfection, Indolicidin, Virus inactivation, BoAHV1

## Abstract

Bovine alphaherpesvirus 1 (BoAHV1) is a reproductively important pathogen whose presence in bovine semen restricts international trade. The increasing use of controlled breeding programs highlights the need for effective viral inactivation strategies. This study evaluated the antiviral activity of the antimicrobial peptide indolicidin in bovine semen experimentally inoculated with BoAHV1. Indolicidin was synthesized by solid-phase peptide synthesis. Antiviral activity and minimum inhibitory concentration (MIC) were determined in MDBK cell cultures. The peptide was then tested in inoculated semen, and semen quality was assessed using computer-assisted sperm analysis (CASA). Indolicidin inhibited BoAHV1 in MDBK cells with an MIC of 8 µM. In semen, viral titer was significantly reduced at 10 µM with complete inactivation at 30 µM, confirmed by the absence of cytopathic effect in MDBK cells. Sperm kinematic, structural and functional integrity analysis demonstrated that semen quality parameters were preserved at concentrations up to 30 µM, while 100 µM caused a significant reduction in total and progressive motility.

## Introduction

Antimicrobial peptides (AMPs) constitute a conserved molecule of the host’s first line of defense, exhibiting broad activity against viruses, bacteria, and fungi, including pathogens resistant to conventional antimicrobial agents (Le et al. 2017). Among these molecules, indolicidin, a cathelicidin peptide isolated from bovine neutrophils, has received particular attention. This 13-residue peptide (ILPWKWPWWPWRR-NH₂) is amphipathic, containing five tryptophan residues that facilitate its interaction with biological membranes. Such structural features support its ability to inhibit a range of viruses as well as Gram-positive and Gram-negative bacteria (Selsted et al. 1992). Although indolicidin has long been recognized for its antiviral activity, important gaps remain regarding its application in bovine semen and its potential effects on sperm motility parameters.

Collected semen is routinely treated with broad-spectrum conventional antibiotics; however, prolonged use of these compounds can impair semen quality (Kumar et al. 2024) and has become increasingly ineffective due to rising antimicrobial resistance. In addition, conventional antibiotics target only bacterial contaminants and provide no control over viruses, which represent a significant concern in reproductive biosecurity (Morrell et al. 2022).

This study investigated the potential of indolicidin to inactivate viruses present in bovine semen. Bovine alphaherpesvirus 1 (BoAHV-1), classified by the World Organisation for Animal Health as a notifiable pathogen of relevance to international trade in animal genetic material (WOAH 2025), was used as the experimental model to evaluate the feasibility of this antiviral approach.

## Material and methods

Indolicidin was synthesized by solid-phase peptide synthesis (SPPS) (Merrifield 1963) and characterized by HPLC and mass spectrometry. Bovine alphaherpesvirus 1 (BoAHV-1; Los Angeles strain) was propagated in Madin–Darby bovine kidney (MDBK) cells cultured in minimal essential medium (MEM) supplemented with 10% fetal bovine serum (FBS). Viral titers were determined using the trimmed Spearman–Karber method (Hamilton et al. 1977). For in vitro antiviral assays, BoAHV-1 suspensions (10⁶·⁷⁵ TCID₅₀/mL) were incubated with indolicidin (4, 6, 8 or 10 µM) for 1 h at room temperature. Tenfold serial dilutions were inoculated into MDBK monolayers in 96-well plates, overlaid with MEM containing 10% FBS, and incubated for 72 h at 37 °C in 5% CO₂. Cytopathic effect (CPE) was evaluated by inverted microscopy, and residual viral titers were calculated. Cell and virus controls were included. 

For semen assays, semen aliquots (500 µL) were experimentally inoculated with BoAHV-1 (10⁵·⁷⁵ TCID₅₀/mL) and incubated with indolicidin (10 or 30 µM) for 1 h at room temperature. Serial dilutions were inoculated into MDBK monolayers, overlaid with MEM containing 5% FBS, and incubated under the same conditions. CPE was assessed after 72 h; samples without CPE underwent three blind passages with freeze/thaw cycles (WOAH 2025; Brunner et al. 1988). The same controls were used.

The effect of indolicidin on spermatozoa was assessed using computer-assisted sperm analysis (CASA) (Amann and Waberski 2014). Semen samples were collected from three Girolando bulls (one per bull) by electroejaculation (Autojac, NEOVET^®^, Uberaba, SP, Brazil) into graduated 15-mL tubes. Immediately after collection, the semen samples were transported to the laboratory and maintained at 37 °C. Only samples exhibiting ≥ 70% total sperm motility were included in the experiments. Subsequently, 500 µL of semen from each bull were aliquoted in triplicate and exposed to indolicidin at concentrations of 30 and 100 µM. The samples were incubated for 15 min at at 37 °C. Following incubation, initial semen quality was assessed according to the guidelines of the Brazilian College of Animal Reproduction (CBRA) (CBRA 2025).

Baseline CASA evaluation (Hamilton-Thorne) was performed using a Makler^®^ chamber (10 µL), assessing total motility, curvilinear velocity (VCL), average path velocity (VAP), straight-line velocity (VSL), straightness (STR), linearity (LIN), beat-cross frequency (BCF) and amplitude of lateral head displacement (ALH). Only samples with total motility ≥ 70% were included. Semen aliquots (500 µL) were incubated with an overestimated concentration of the peptide (30 or 100 µM) for 15 min and reanalyzed using the same CASA protocol. Data were analyzed using analysis of variance followed by Tukey’s test in R (R Core Team 2025) (*p* < 0.05) and are presented as mean ± SD.

For plasma membrane, acrosome, and chromatin integrity assessments, frozen semen straws from three bulls with proven fertility in vitro embryo production programs were thawed at 37 °C for 30 s. For each bull, three semen straws were thawed. The semen from the three straws was pooled into a single sample, transferred to 1.5-mL microcentrifuge tubes, divided into 150-µL aliquots, and incubated in duplicate under the experimental conditions (30 µM of peptide for 30 min at 37 °C).

Only samples with progressive motility ≥ 60% and vigor score 3 immediately after thawing were included. Three smears were prepared immediately after thawing and after incubation; one was stained with eosin–nigrosin, whereas the remaining two were used for subsequent analyses. Plasma membrane integrity was evaluated by eosin–nigrosin supravital staining. Briefly, 10 µL of semen was mixed with 10 µL of stain, air-dried, and examined under an Olympus BX41 light microscope (100× oil immersion). One hundred spermatozoa were evaluated per smear (Agarwal et al. 2016). To confirm that indolicidin itself, given its membrane-disrupting mechanism of action, does not compromise sperm membrane integrity, an additional set of three bulls was evaluated by the same eosin–nigrosin staining protocol.

Acrosome integrity was assessed using fluorescein isothiocyanate-conjugated (FITC-PSA). Smears were fixed in methanol (− 10 °C, 30 s), air-dried, incubated with 60 µL FITC-PSA for 20 min, washed with phosphate-buffered saline (PBS), and examined under UV fluorescence microscopy (100×). Two hundred spermatozoa were classified as having intact (uniform green fluorescence) or reacted/damaged acrosomes (adapted from Fagundes et al. 2010).

Chromatin integrity was evaluated using the acridine orange assay. Smears were fixed overnight in Carnoy’s solution (ethanol acid, 3:1), stained with acridine orange according to Tejada et al. (1984), and examined after 5 min. Two hundred spermatozoa were evaluated per smear; green fluorescence indicated intact chromatin, whereas red, orange, or intermediate fluorescence indicated partial or complete chromatin denaturation (Tejada et al. 1984).

## Results

### In vitro antiviral activity of the peptide against BoAHV1

**Fig. 1:**
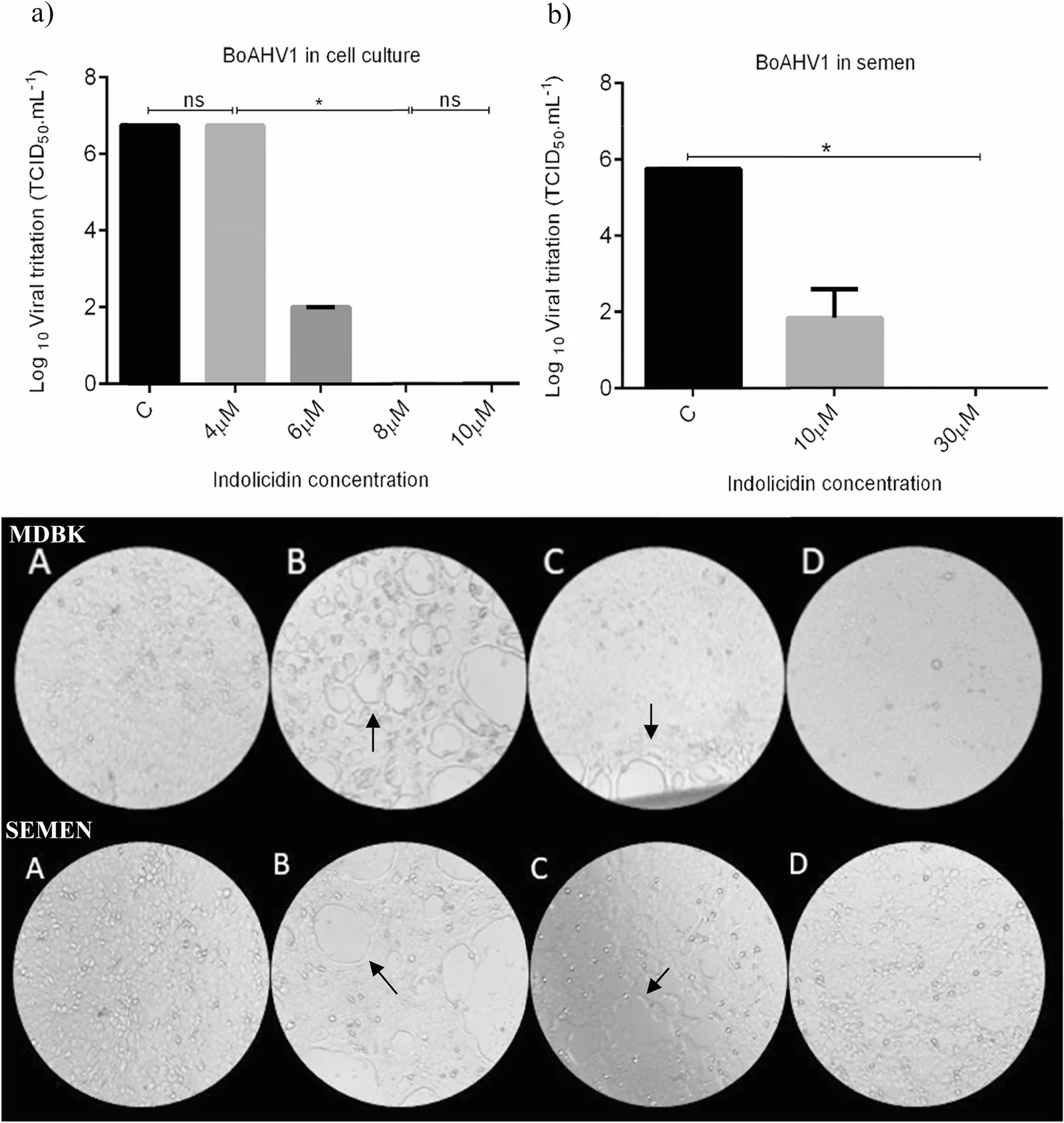
(**a**) viral titers as a function of indolicidin concentration in MDBK cell culture and (**b**) in bovine semen experimentally inoculated with BoAHV-1. (**c**) MDBK panel: optical microscopy (20X) showing the effect of indolicidin on the viability of the BoAHV1 in MDBK cell culture. (**A**) uninfected control (no CPE); (**B**) positive control with BoAHV1 without peptide (CPE); (**C**) BoAHV1 treated with 6 µM of peptide (lower CPE); (**D**) BoAHV1 treated with 8 µM of peptide (no CPE); SEMEN panel: MDBK culture showing the effect of indolicidin on the viability of the BoAHV1 in the semen. (**A**) uninfected control (no CPE). (**B**) positive control with BoAHV1 without peptide (CPE). (**C**) treated with 10 µM of peptide (low CPE). (**D**) treated with 30 µM of peptide (no CPE). Arrows: typical grape-like clusters of rounded cells gathered, characteristic of the CPE caused by BoAHV1 infection. *P values < 0.05 were considered statistically significant

Indolicidin achieved complete inactivation of BoAHV-1 in MDBK cells at minimum inhibitory concentration (MIC) of 8 µM (Fig. 1a) as also indicated by the absence of the characteristic CPE (Fig. 1c - MDBK panel D). At 6 µM, the peptide did not fully prevent infection but produced a marked reduction in viral titer (> 4 log₁₀) compared with the untreated control. This reduction in infectivity is evident when contrasting the residual CPE in the 6 µM treatment (Fig. 1c - MDBK panel C) compared with the widespread CPE observed in the positive control (Fig. 1c - panel MDBK B), typically characterized by the grape-like clusters of rounded cells gathered (arrows), characteristic of the cytopathic effect caused by BoAHV1 infection (WOAH 2025).

### In vitro antiviral activity of the peptide in semen inoculated with BoHV-1

After confirming the antiviral effect of indolicidin in cell culture, the peptide was evaluated in bovine semen experimentally inoculated with BoAHV-1. Unlike the findings in MDBK monolayers, where complete viral inactivation occurred at 8 µM, treatment of inoculated semen required a MIC of 10 µM to achieve a reduction of nearly 4 log₁₀ in viral titer compared with the untreated semen control (Fig. 1b). At this concentration, however, residual infectivity was still detected following three blind passages in MDBK cells (Fig. 1c - SEMEN panel C). For this reason, a higher, broadly effective concentration (30 µM) was tested. At 30 µM, indolicidin completely inactivated BoAHV-1 (Fig. 1b), also demonstrated by the absence of CPE in MDBK cells after serial passage (Fig. 1c - SEMEN panel D) compared with the extensive CPE observed in the positive control (Fig. 1c - SEMEN panel B). Notably, MDBK monolayers exposed to semen treated with this peptide concentration displayed normal cell growth patterns, indicating that indolicidin at 30 µM did not exert cytotoxic effects under the conditions evaluated.

### Effect of indolicidin on bovine semen

To confirm the absence of adverse effects of indolicidin on bovine semen, the MIC (30 µM) identified in the in vitro antiviral activity in inoculated semen assay was assessed together with an intentionally high peptide concentration (100 µM) (Table 1). Treatment with 30 µM indolicidin showed no significant differences in any parameter when compared with the untreated control, indicating that this concentration did not impair sperm quality. Interestingly, at high concentration (100 µM), indolicidin did not significantly affect VAP, VSL, VCL, ALH, STR or LIN values relative to the control group. Although BCF increased at 100 µM, this value remained within the range observed for the control. Despite maintaining most kinematic standards at this concentration, a significant reduction in total and progressive motility (MT and MP) was detected, rendering semen treated at 100 µM unsuitable for insemination.

**Table 1:** Effect of indolicidin (30 and 100 µM) on sperm kinematic parameters in fresh semen from three bulls after 15 min of incubation at 37 °C, assessed by computer-assisted sperm analysis (CASA)

Semen quality parameters	Control	Indolicidin 30 µM	Indolicidin 100 µM
MT (%)	79.3 ± 2.9^a^	76.3 ± 2.1^a^	26.7 ± 10.6^b^
MP (%)	37.7 ± 9.1^a^	34.7 ± 11.6^a^	12.7 ± 4^b^
VAP (µm/s)	114.9 ± 34.8^a^	120.3 ± 41.4^a^	114.3 ± 37.6^a^
VSL(µm/s)	76.6 ± 15.8^a^	76.4 ± 14.8^a^	74.1 ± 11^a^
VCL(µm/s)	205.8 ± 73.1^a^	215.8 ± 85.8^a^	209.5 ± 80.2^a^
ALH (µm)	8.3 ± 2.1^a^	8.5 ± 2.6^a^	7.3 ± 1.8^a^
BCF (Hz)	33.1 ± 5.1^ab^	32 ± 4.9^b^	36 ± 5.2^a^
STR (%)	71.3 ± 10.6^a^	67 ± 11.3^a^	70 ± 10.7^a^
LIN (%)	40.3 ± 7.8^a^	40 ± 10.4^a^	43 ± 8.5^a^

Means followed by the same lowercase letter between columns do not differ significantly from each other (*p*-value < 0.05). *MT* Total motility, *MP* Progressive motility, *VAP* Average path velocity, *VSL* straight-line velocity, *VCL* curvilinear velocity, *ALH* amplitude of lateral head displacement, *BCF* beat cross frequency, *STR* Straightness, *LIN* Linearity

Moreover, sperm membrane integrity, acrosome integrity, and chromatin damage were evaluated using only the 30 µM concentration of indolicidin. As in the CASA analysis, we deliberately challenged the experimental conditions by pre-incubating the samples with the peptide for 30 min immediately after thawing to determine whether the peptide would remain innocuous under more stringent conditions, beyond those normally employed in reproductive procedures. As shown in Table 2, none of the treated samples differed significantly from the corresponding controls in the absence of the peptide.

**Table 2:** Effects of indolicidin (30 µM) on sperm plasma membrane and acrosome integrity and chromatin damage in thawed bovine semen from three bulls following 30 min of incubation at 37 °C

Semen quality parameter (%)	Bull 1		Bull 2		Bull 3	
	Control	Indolicidin 30 µM	Control	Indolicidin 30 µM	Control	Indolicidin 30 µM
Membrane integrity	64.2 ± 3.0^a^	68.5 ± 4.0^a^	72.4 ± 17.0^a^	81.3 ± 17.2^a^	72.8 ± 0.6^a^	76.8 ± 7.2^a^
Acrosome integrity	42.7 ± 1.6^a^	56.6 ± 1.6^a^	40.7 ± 1.9^a^	51.6 ± 1.0^a^	41.0 ± 1.0^a^	52.4 ± 1.0^a^
Chromatin damage	7.7 ± 0.6^a^	5.1 ± 0.5^a^	10.7 ± 0.9^a^	5.6 ± 0.7^a^	4.5 ± 0.5^a^	5.3 ± 0.5^a^

Means followed by the same lowercase letter between columns do not differ significantly from each other (*P* value < 0.05)

## Discussion

Different mechanisms of action previously described for indolicidin may help explain the peptide activity against BoAHV-1 in this study. Falla et al. (1996) reported that, in Gram-negative bacteria, the peptide is able to traverse the outer membrane and induce disruption of the plasma membrane. In contrast, Subbalakshmi et al. (2000) did not observe membrane lysis in *E. coli*, but instead documented an increase in membrane permeability that facilitated peptide entry, followed by inhibition of DNA and protein synthesis within the cytoplasm.

Given that BoAHV-1 consists of an icosahedral capsid covered by a host-derived lipid bilayer envelope (Oirschot 1995), it is plausible that these mechanisms could act in combination. Glycoproteins present in the viral envelope are directly responsible for the adsorption of the virus to specific cell receptors present in the host cell membrane during the initial stages of infection (Muylkens et al. 2007). Therefore, direct disruption of the viral envelope would provide a virucidal effect, compromising the structural integrity of the virion, whereas interaction of indolicidin with viral DNA could exert an intracellular antiviral effect, preventing genome replication after entry into host cells. Together, these complementary actions offer a possible explanation for the marked reduction in BoAHV-1 infectivity observed in our assays.

When comparing the MIC of indolicidin in culture medium with that required in bovine semen, a marked increase in the concentration needed for complete viral inactivation was evident. This discrepancy is likely due to the complex biochemical composition of semen, which contains carbohydrate, proteins, lipids and other biomolecules capable of binding or sequestering antimicrobial peptides. Such interactions may reduce the amount of free, biologically active indolicidin available to interact with viral targets, thereby requiring higher concentrations to achieve effective BoAHV-1 inactivation. This matrix effect offers a plausible explanation for the MIC adjustment (30 µM) required in the semen applications.

Although complete viral inactivation was achieved with 30 µM indolicidin, it is important to consider that the viral titer used for experimental contamination in this study (10⁵·⁷⁵ TCID₅₀/mL) was intentionally high and exceeds the levels typically detected in naturally infected semen. Considering that natural infections generally present titers around 10² TCID₅₀/mL (Flores et al. 2005), it is reasonable to assume that 10 µM indolicidin could be sufficient for complete disinfection, as this concentration reduced the viral titer by approximately 4 logs.

Sperm motility remains a key parameter for evaluating semen quality, as motile spermatozoa are essential for reaching the female reproductive tract and achieving fertilization. Although all motility parameters were maintained when 30 µM of indolicidin was used for semen disinfection, an overestimated peptide concentration (100 µM) was considered detrimental to sperm viability, as a minimum motility threshold of 50% is desirable for acceptable reproductive performance. Compared to the control, the significant reduction in straight and progressive motility observed at this concentration renders its use impractical for insemination.

In addition, indolicidin did not exert detectable deleterious effects on sperm quality, as membrane integrity, acrosome integrity, and chromatin integrity remained comparable to those observed in the untreated controls, even after prolonged incubation (Table 2). These findings suggest that the peptide is well tolerated by sperm cells under the experimental conditions evaluated. Interestingly, although no statistically significant differences were detected, indolicidin-treated samples consistently showed a modest trend toward improved values for all assessed sperm quality parameters relative to the controls. While this tendency should be interpreted with caution, it indicates that indolicidin does not compromise sperm function and may even contribute to maintaining sperm quality during incubation. However, further studies under conditions that more closely resemble practical application, including functional fertility assessments such as in vitro fertilization (IVF) or related fertilization assays, are warranted to determine whether this apparent beneficial effect is translatable to field conditions.

Alternative and innovative approaches for the decontamination of bovine semen, such as antimicrobial photodynamic inactivation, have recently been reported (Oliveira et al. 2020, 2024). Likewise, the antimicrobial activity of bioactive peptides is well established (Vasilchenko et al. 2017; Shagaghi et al. 2016). However, despite their recognized antimicrobial potential, the use of indolicidin and its derivatives in semen disinfection still remains incipient. Only recently, Metz et al., (2025) demonstrated that indolicidin effectively inactivated Equine Arteritis Virus in semen while preserving sperm motility and vigor (Metz et al. 2025). A recent review by Kumar et al. (2024) offers a comprehensive overview of the origin and potential application of AMPs in the semen of several species, including peptides in boar semen (hexapeptides c-WFW (10 µM) and c-WWW (20 µM), PMAP-37 peptide (3µM) and bacteriocin (1%), nisin peptide (50 µg/mL) in rat, LL-37 (10 µM) peptide in goat and a human β-defensin (10 µg/mL). Even less explored is the application of such peptides in bovine semen. Sánchez-Acosta et al. (2020) demonstrated that dermaseptin S4 derivatives (15 µM) exhibited in vitro antibacterial activity without impairing sperm motility, however, no previous study has assessed the use of AMPs for viral inactivation in semen. Therefore, indolicidin emerges as a promising candidate to address this gap, offering a feasible alternative for broadening the range of strategies applicable to semen preservation.

Previous studies have demonstrated that indolicidin exhibits bactericidal activity at concentrations up to 32 µM (Vergis e al. 2019). In this context, the complete viral inactivation observed in semen at 30 µM in the present study is particularly significant. This finding suggests that, at this concentration, indolicidin may act as a broad-spectrum disinfectant, potentially enabling simultaneous antimicrobial activity in a single-step treatment. Such an approach could help reduce or even eliminate the use of conventional antibiotics in semen extenders, thereby contributing to antimicrobial management. Moreover, the methodology employed here is readily adaptable to routine procedures in semen collection and processing centers, offering a favorable cost-benefit profile.

Indolicidin also possesses physicochemical features of interest for industrial application. Its short length allows high yield synthesis, while the feasibility of recombinant production offers a cost-effective route for large scale manufacturing, supporting its potential commercial use.

Despite BoAHV-1 being used exclusively in this study as a model virus to evaluate the disinfectant potential of indolicidin, the peptide’s mode of action indicates that its applicability may extend beyond this pathogen. In addition to bacteria, several other viruses of reproductive importance are enveloped, and indolicidin’s known membranolytic and intracellular activities suggest that it may exert enhanced antiviral effects against this broader group of agents. This raises the prospect that indolicidin could serve as a versatile tool for controlling a range of semen-borne pathogens in reproductive technologies.

## Conclusion

Indolicidin demonstrated marked in vitro antiviral activity against BoAHV-1, achieving complete viral inactivation in cell culture and experimentally inoculated bovine semen. A concentration of 30 µM was required for full inactivation in semen, likely due to matrix effects, and did not impair sperm kinematic, structural and functional integrity parameters, whereas higher concentrations negatively affected sperm motility. These findings indicate that indolicidin can effectively inactivate BoAHV-1 in semen under experimental conditions while preserving sperm quality. This peptide represents a promising alternative to conventional antimicrobial strategies in semen processing and warrants further investigation under field and fertility conditions.

## Data Availability

No datasets were generated or analysed during the current study.
